# Identification of potential biomarkers related to glioma survival by gene expression profile analysis

**DOI:** 10.1186/s12920-019-0479-6

**Published:** 2019-03-20

**Authors:** Justin Bo-Kai Hsu, Tzu-Hao Chang, Gilbert Aaron Lee, Tzong-Yi Lee, Cheng-Yu Chen

**Affiliations:** 1 Department of Medical Research, Taipei Medical University Hospital, Taipei, 110 Taiwan; 2 Graduate Institute of Biomedical Informatics, Taipei Medical University, Taipei, 110 Taiwan; 3 Warshel Institute for Computational Biology, The Chinese University of Hong Kong, Shenzhen, 518172 China; 4 School of Science and Engineering, The Chinese University of Hong Kong, Shenzhen, 518172 China; 5 School of Life and Health Science, The Chinese University of Hong Kong, Shenzhen, 518172 China; 6 Research Center of Translational Imaging, College of Medicine, Taipei Medical University, Taipei, 110 Taiwan; 7 Department of Radiology, School of Medicine, College of Medicine, Taipei Medical University, Taipei, 110 Taiwan; 8 Department of Medical Imaging and Imaging Research Center, Taipei Medical University Hospital, Taipei Medical University, Taipei, 110 Taiwan; 9 Department of Radiology, Tri-Service General Hospital, Taipei, 114 Taiwan; 10 Department of Radiology, National Defense Medical Center, Taipei, 114 Taiwan

**Keywords:** Low-grade glioma (LGG), High-grade glioma, Gene signature, Biomarkers, Prognosis

## Abstract

**Background:**

Recent studies have proposed several gene signatures as biomarkers for different grades of gliomas from various perspectives. However, most of these genes can only be used appropriately for patients with specific grades of gliomas.

**Methods:**

In this study, we aimed to identify survival-relevant genes shared between glioblastoma multiforme (GBM) and lower-grade glioma (LGG), which could be used as potential biomarkers to classify patients into different risk groups. Cox proportional hazard regression model (Cox model) was used to extract relative genes, and effectiveness of genes was estimated against random forest regression. Finally, risk models were constructed with logistic regression.

**Results:**

We identified 104 key genes that were shared between GBM and LGG, which could be significantly correlated with patients’ survival based on next-generation sequencing data obtained from The Cancer Genome Atlas for gene expression analysis. The effectiveness of these genes in the survival prediction of GBM and LGG was evaluated, and the average receiver operating characteristic curve (ROC) area under the curve values ranged from 0.7 to 0.8. Gene set enrichment analysis revealed that these genes were involved in eight significant pathways and 23 molecular functions. Moreover, the expressions of ten (*CTSZ, EFEMP2*, *ITGA5*, *KDELR2*, *MDK*, *MICALL2, MAP 2 K3*, *PLAUR*, *SERPINE1*, and *SOCS3*) of these genes were significantly higher in GBM than in LGG, and comparing their expression levels to those of the proposed control genes (*TBP*, *IPO8*, and *SDHA*) could have the potential capability to classify patients into high- and low- risk groups, which differ significantly in the overall survival. Signatures of candidate genes were validated, by multiple microarray datasets from Gene Expression Omnibus, to increase the robustness of using these potential prognostic factors. In both the GBM and LGG cohort study, most of the patients in the high-risk group had the *IDH1* wild-type gene, and those in the low-risk group had *IDH1* mutations. Moreover, most of the high-risk patients with LGG possessed a 1p/19q-noncodeletion.

**Conclusion:**

In this study, we identified survival relevant genes which were shared between GBM and LGG, and those enabled to classify patients into high- and low-risk groups based on expression level analysis. Both the risk groups could be correlated with the well-known genetic variants, thus suggesting their potential prognostic value in clinical application.

**Electronic supplementary material:**

The online version of this article (10.1186/s12920-019-0479-6) contains supplementary material, which is available to authorized users.

## Background

Glioma is a common type of primary central nervous system (CNS) tumor which arises from glial cells [1]. Following the World Health Organization (WHO) classification in 2007, gliomas can be subdivided into grade II, grade III, and grade IV (glioblastoma multiforme, GBM), depending on the degree of aggressiveness [2, 3]. In “The Cancer Genome Atlas” (TCGA) database, grade II and III are classified as lower-grade glioma (LGG), and grade IV as GBM. Despite developments in therapies that include surgical resection, chemotherapy, and radiotherapy, the median survival and prognosis remain poor, particularly for glioblastoma patients [4, 5]. The median overall survival time (mOS) of GBM is approximately 1.25 years [5, 6], and that of LGG is 6.5–8 years [7, 8]. Thus, it is important to elucidate the survival events of glioma, which could potentially aid in the diagnosis and prognosis of glioma patients.

Patient survival time with regards to tumor progression is associated with various subtypes and grades of the tumor [2]. The histological classification of tumor subtypes is important to guide treatment decisions, which are often combined with several clinical prognostic features. In neuro-oncological practice, however, no clear national consensus for adult glioma diagnosis has been reached and the diagnosis is subject to interobserver variation [9, 10]; only utilizing histological information in studying various types of gliomas is restricted. On the other hand, previous studies have shown that gene expression profiling provides an objective method to classify tumors [11, 12]; it is better to correlate gene expression profiling, rather than tumor histology, with prognosis [13]. Moreover, it may even be utilized to predict patients’ prognosis from various points of view [14–29]. Comparing these gene lists published from 2004 to 2016, it is observed that the genes identified from various research groups are quite different. This observation indicates that glioma patients’ overall survival (OS) is correlated with many kinds of events caused by various expression profiles of multiple genes. Therefore, extraction of comprehensive survival-related genes associated with gliomas is required, and it is possible for researchers to carry out further relevant studies. In addition, most previous studies [14, 18–23, 25–27, 29] have only utilized microarray datasets, rather than different kinds of datasets such as next-generation sequencing (NGS) data, to screen expression profiles of genes might have unexpected data bias; generally, utilizing NGS to detect gene signatures might be more precise than array data.

In this study, we aimed to identify common genes correlated with the overall survival of gliomas following the association of their expression profiles and patients’ survival time. Candidate genes were extracted from GBM and LGG study cohorts after analysis of NGS datasets from TCGA and validation by microarray datasets from Gene Expression Omnibus (GEO). Of these survival-related genes, the critical ones, which were potential biomarkers, were further analyzed and filtered, and then used to construct the survival-relevant risk models for clinical application against gliomas.

## Methods

### Patients and gene expression datasets

Publicly available gene expression datasets of patients with glioma were obtained from TCGA (https://cancergenome.nih.gov/) and the GEO (https://www.ncbi.nlm.nih.gov/geo/). From TCGA projects (TCGA-LGG and TCGA-GBM), level 3 RNA-Seq datasets and their clinical information were used to investigate the relationship between gene expression and patient survival. The microarray datasets (GSE16011, GSE4412, and GSE4271) from the GEO were utilized to confirm and validate the results obtained based on the TCGA datasets. Notably, grades II and III of gliomas were included in the TCGA-LGG project, whereas grade IV was studied in a separate project, namely TCGA-GBM. Following the project definition, patients from GEO datasets could be divided into LGG and GBM categories. The sample sizes of various datasets are summarized in Table 1, and the detailed clinical and histological characteristics of patients are listed in Table 2.

**Table 1:** Statistics of datasets from TCGA and GEO databases

	TCGA (RNA-Seq)	GEO GSE16011)	GEO (GSE4412)	GEO (GSE4271)
GBM	154	159	59	76
LGG	516	109	26	24
GBM with surv. Info.	152	155	59	56
LGG with surv. Info.	511	109	26	21

When the analysis of gene expression must be correlated to survival information, the sample size of datasets would be possible to become smaller

**Table 2:** Clinical and histological characteristics of patients with glioma

	TCGA (RNA-Seq)		GSE16011		GSE4412		GSE4271	
	LGG	GBM	LGG	GBM	LGG	GBM	LGG	GBM
	516	154	109	159	26	59	24	76
Sex								
Male	285	99	72	108	6	26	16	52
Female	230	54	37	51	20	33	8	24
Unknown	1	1						
Age								
Median (LQ-UQ)	41 (32–53)	60 (52–70)	44 (37–55)	55 (46–64)	34 (31–41)	47 (39–61)	35 (32–43)	49 (40–55)
Histology								
Astro.	194 (37.6%)		29 (24.8%)		8 (30.8%)			
GBM		154 (100%)		159 (100%)		59 (100%)		
Oligoastro.	130 (25.2%)		28 (23.9%)		7 (26.9%)			
Oligodendro.	191 (37.0%)		52 (44.4%)		11 (42.3%)			
Pilocytic Astro.								
Unknown	1 (0.2%)							
Tumor grade								
G1								
G2	249 (48.3%)		24 (20.5%)					
G3	266 (51.6%)		85 (73.7%)		26 (100%)		24 (100%)	
G4		153 (99.4%)		159 (100%)		59 (100%)		76 (100%)
Unknown	1 (0.1%)	1 (0.6%)						

**Fig. 1:**
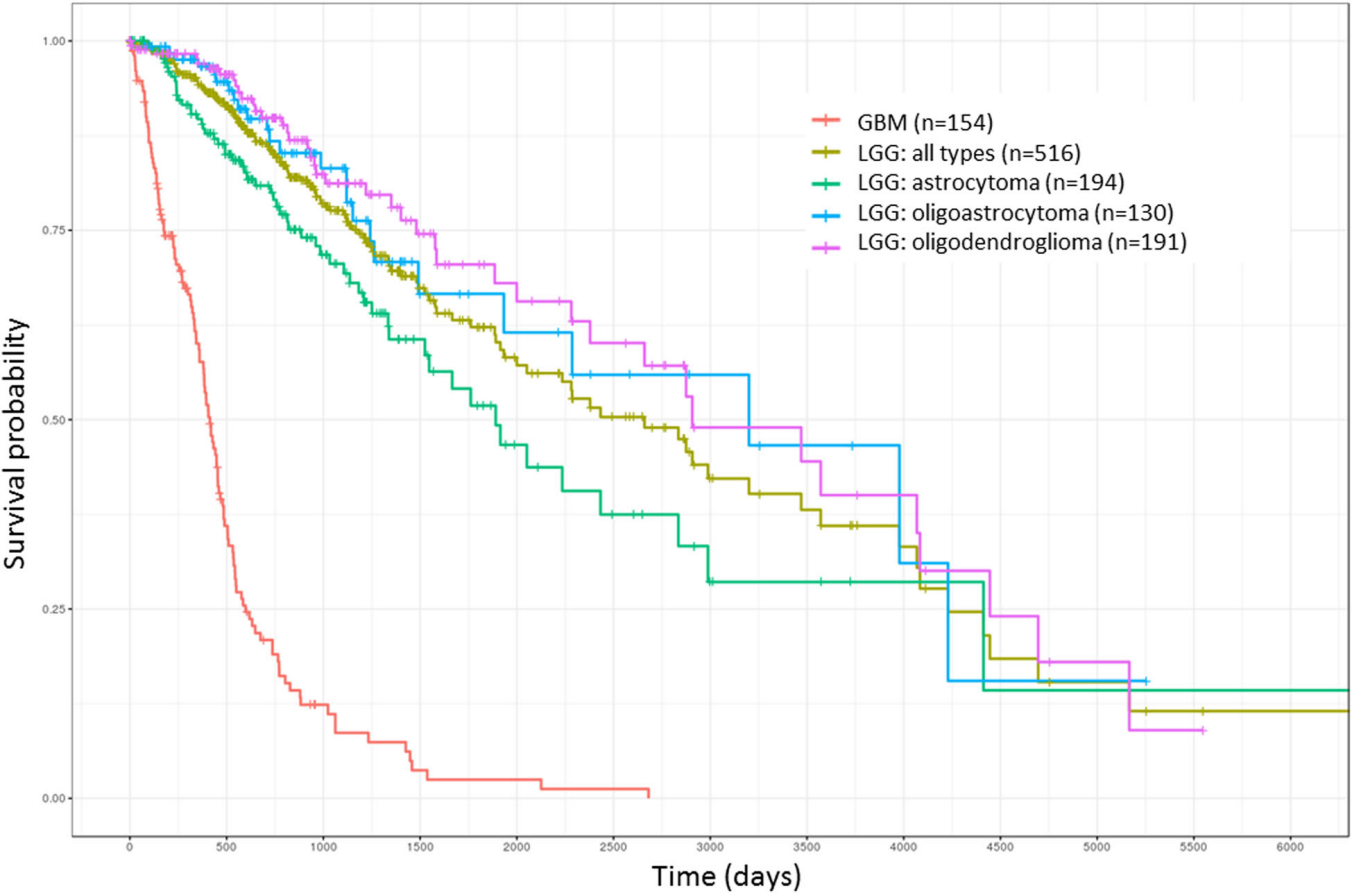
OS curve of various histological subtypes of gliomas (TCGA samples). LGG was divided into three subtypes: astrocytoma, oligoastrocytoma, and oligodendroglioma. X-axis: patients’ OS duration (days); Y-axis: patients’ survival rate

In this study, NGS datasets were used for the main analysis because of their advantages of low data bias and large sample size. The median OS of patients with gliomas based on their various histological subtypes was estimated using the Kaplan-Meier curve (Fig. 1). The median OS of patients with LGG and those with GBM were determined as approximately 2700 and 450 days, respectively.

The level-3 data (RNA-Seq) obtained from TCGA utilized the fragments per kilobase of transcript per million mapped reads (FPKM) [30] to determine the expression level of genes. The formula for FPKM is as follows:

$$ \mathrm{FPKM}=\frac{total\ fragments}{mapped\ reads\ (millions)\ast exon\ length\ \left( kilobase\ pair\right)} $$

After exclusion of genes that were not expressed in all patients, 19,924 genes were eligible for further analysis of the LGG and GBM cohorts from TCGA projects. Gene expression analysis of microarrays belonging to the GBM and LGG populations from the GEO database were first normalized using the R function *normalize.quantiles* [31].

### Analysis workflow of this study

**Fig. 2:**
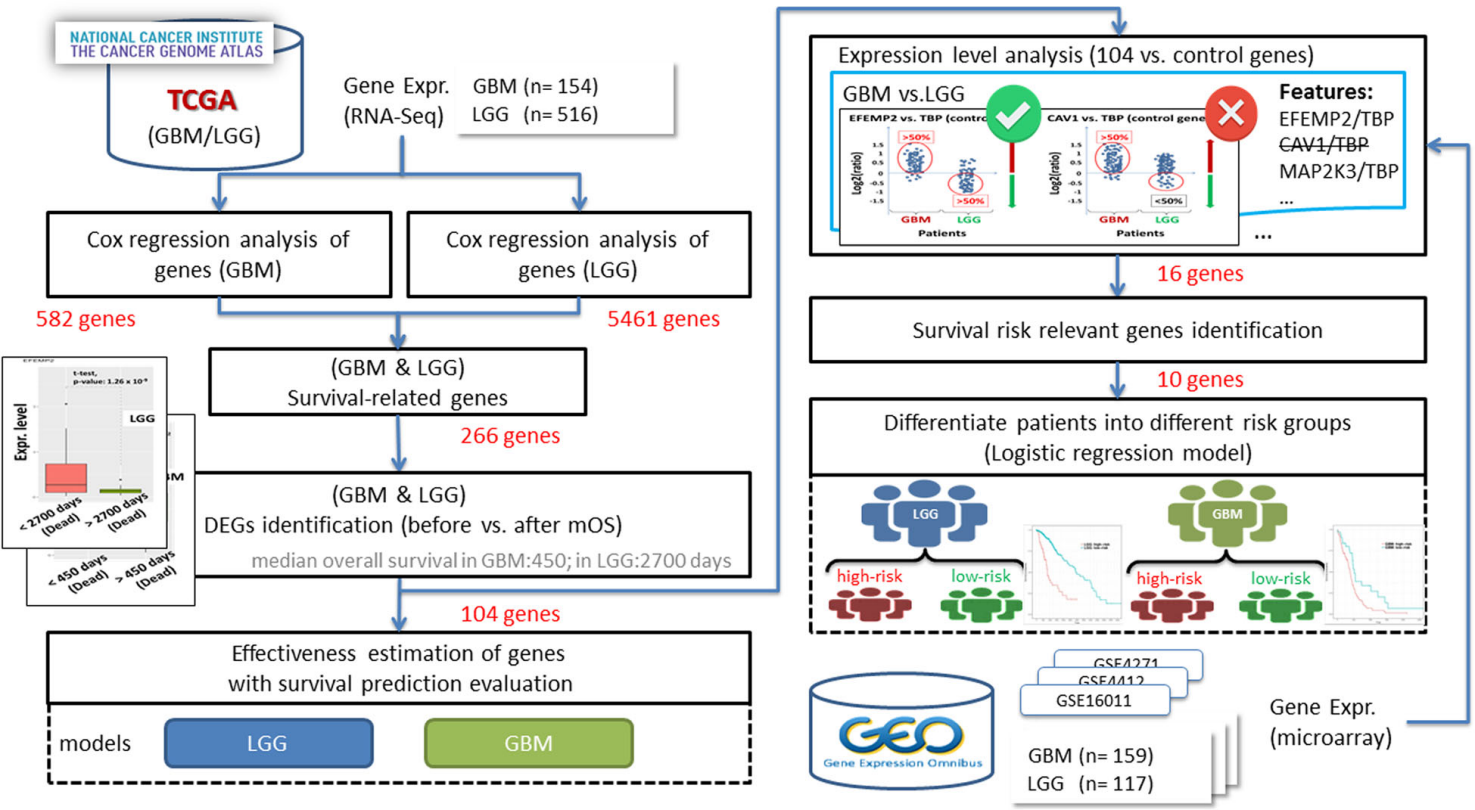
System workflow. The left-hand-side figure was to identify the shared survival-related genes from LGG and GBM. The gene set was scaled down against a series analysis method. Then, the importance of candidate genes was proved through the performance estimation of survival predictors. The right-hand-side figure shows the extraction of survival-relevant biomarker representatives from these genes, which could be used in clinical practice

This study was divided into two major parts. In the first part, survival-related genes were identified and their effectiveness in relation to the survival of patients with GBM and LGG was evaluated. In the second part, a representative subset of these genes that could help in differentiating between high- and low-risk patients was identified (Fig. 2). The first part focused on profiling gene signatures that corresponded with the patients’ OS; these genes were termed survival-related genes. The performance evaluation of survival predictors for GBM and LGG indicated that these genes were closely correlated with patient outcomes (survival time). Subsequently, gene set enrichment analysis using various tools was performed on these genes (not shown in the Fig. 2). In the second part, we used additional microarray datasets to further filter genes whose expression trends between the LGG and GBM groups were consistent with the results of the analysis of the NGS datasets and may have been applicable for classification of patients into risk groups (high-risk patients have shorter OS; low-risk patients have longer OS). The size of the gene set was gradually scaled down and is annotated in Fig. 2 after several filtering steps using various criteria.

### Identification of significant survival-related genes

The Cox proportional hazards regression model (“Cox model” hereafter; survival analysis) was used to identify possible factors that might be associated with patients’ OS duration. In this study, univariate Cox regression analysis [32] was performed to assess the expression profiles of genes that might be significantly correlated with the survival time of patients with GBM or LGG. Subsequently, these putative survival-related genes were ranked and filtered by applying stringent criteria (hazard ratio [HR] > 1; Wald test, *p* < 0.01). Each extracted gene was consequently analyzed to evaluate the correlation of its expression level with various survival durations in patients. Here, the median OS (in days) of GBM and LGG groups would be set as an important time point for both groups, respectively, to separate patients into shorter or longer survival durations, to recognize that the expression levels of genes differed significantly between the survival durations (number of days to death less or more than the median OS). The Student’s *t*-test (*p* < 0.05) was conducted to select statistically significant candidate genes.

### Building survival predictive models for patients with GBM and LGG

The predictive model for survival analysis in this study was built using *randomForestSRC* [33–35], a nonparametric machine learning method. Moreover, because it can combine the results of many survival trees, this model is arguably more objective than other methods. Accordingly, the expression profiles of candidate genes related to survival durations were used to construct survival predictors for GBM and LGG. To assess the performance of the predictors, 1000 repetitions of five-fold cross-validation were performed, 80% of the samples were employed as the training dataset to train the model, and the remaining 20% served as the validation dataset. Receiver operating characteristic curves (ROC) obtained from the 1000 iterations were evaluated using a boxplot with their area under curve (AUC) values. The performance could be used to realize the importance of these candidate genes to GBM and LGG.

### Gene set enrichment analysis

Ingenuity pathway analysis (IPA) software (Qiagen), GeneAnalytics [36], and DAVID [37, 38] were used to analyze the biological roles and molecular functions of candidate genes identified from patients with glioma. Survival-related genes common to both LGG and GBM could be useful in realizing shared functions; the pathways in both study cohorts were related to patient survival.

In this study, multiple gene set enrichment analysis tools were applied to increase the consistency and accuracy of the results. The functions and pathways that the gene set was involved were identified using at least two kinds of tools.

### Gene expression level analysis between GBM and LGG

The survival-related genes with varying expression levels in case of relative high-risk (GBM) and relative low-risk (LGG) of gliomas would be further analyzed and could be used as putative biomarkers. A previous study demonstrated that the following five endogenous control genes were not differentially expressed between the glioma and normal brain: *TBP*, *IPO8*, *GAPDH*, *RPL13A*, and *SDHA* [39]. Therefore, the log2-fold changes in the expression of these survival-related genes relative to those of the control genes were calculated; there were *p* × *q* unique features (the signatures of genes were higher or lower than those of the control genes) for each patient, when *p* survival-related genes and *q* control genes were present. For each feature, the percentages of patients with high and low expression were calculated and screened. If a gene expression was both high (or low) in over 50% of patients with GBM and low (or high) in over 50% of those with LGG, compared with the expression of the control genes, the log2-fold change value was used as a feature in this study. Subsequently, features with different expression levels between GBM and LGG were retained as the candidates of risk descriptors. For instance, the survival-related gene *TIMP1*- which had high expression in 98% of patients with GBM but low expression in 60% of patients with LGG compared with the reference gene TBP - was retained. In addition to RNA-Seq datasets (TCGA), three distinct microarray datasets (GEO) were utilized to validate the consistency of various gene signatures in both classes of patients, in order to increase the data strength.

### Survival risk relevant genes identification

The median OS days of GBM and LGG were notably different, implying that patients with GBM have a shorter survival time (relative to high-risk) and those with LGG have longer survival time (relative to low-risk). Under this assumption, survival-related genes were first filtered (using the method mentioned in the previous section) as possible descriptors to classify patients into risk groups. However, the effectiveness of these genes needed to be determined for further analysis using a statistical model. A logistic regression model (*Y* = *X*_1_ × *β*_1_ + *X*_2_ × *β*_2_ +  …  + *X*_*n*_ × *β*_*n*_ + *k*) was applied to evaluate the importance of these features, namely the survival-related genes versus the control genes. Here, *Y* is the estimated value of glioma prognosis risk (GBM defined as 1, LGG defined as 0), *X* represents the value of the log2-fold change of each feature, β is the unknown coefficient, and *k* is the unknown constant. The Akaike information criterion (AIC) was utilized to evaluate the relative quality of all models, which were constructed with various combinations of features. While repeating the process (backward elimination) to construct the logistic regression model, features with low predictive value for glioma prognosis were excluded each time until the number of features that provided the smallest AIC values was reached. Consequently, these features would be capable of recognizing the survival risk of patients with GBM or LGG; thus, the expression level of those genes relative to that of the control genes could be correlated to patients’ survival.

### Differentiation of patients into different risk groups

After the candidate features (from previous section: Survival risk relevant genes identification) had been identified, they could be used directly to create risk models for GBM and LGG. Logistic regression was applied to construct both risk models. Here, the outcome variable *Y* was the estimated GBM or LGG prognosis risk (patients can’t live over mOS are relative high risk and can live longer than mOS are low risk); for the GBM risk model, survival durations shorter than 450 days were defined as 1 and those longer than 450 days are defined as 0. Similarly, for the LGG risk model, survival durations shorter than 2700 days were defined as 1 and those longer than survival durations longer than 2700 days were defined as 0. The variable *X* would be substituted into the log2-fold change value of the candidate features. The other variables, such as β and *k* were then estimated with the R package generalized linear models (glm) function for GBM and LGG risk model, respectively. In addition, 1000 repetitions of the five-fold cross-validation were run to evaluate the GBM and LGG models, which were used to classify patients into different risk groups.

## Results

### GBM and LGG shared key survival-related genes

In this study, using gene expression profiling, we identified 104 genes that were significantly correlated with OS in patients with GBM and those with LGG. After application of the stringent criteria to filter the putative survival-related genes using the Cox model, the expression signatures of 582 and 5461 genes were identified and correlated to OS in case of GBM (*n* = 152) and LGG (*n* = 511), respectively. Subsequently, 266 genes were obtained through the gene lists from both study cohorts. However, only 104 of these genes were also significantly differentially expressed (t-test, *p* < 0.05) before and after the median OS time in the GBM and LGG study cohorts; these 104 survival-relevant genes are listed in Additional file 1: Table S1.

### Effectiveness estimation of 104 genes for GBM and LGG survival

**Fig. 3:**
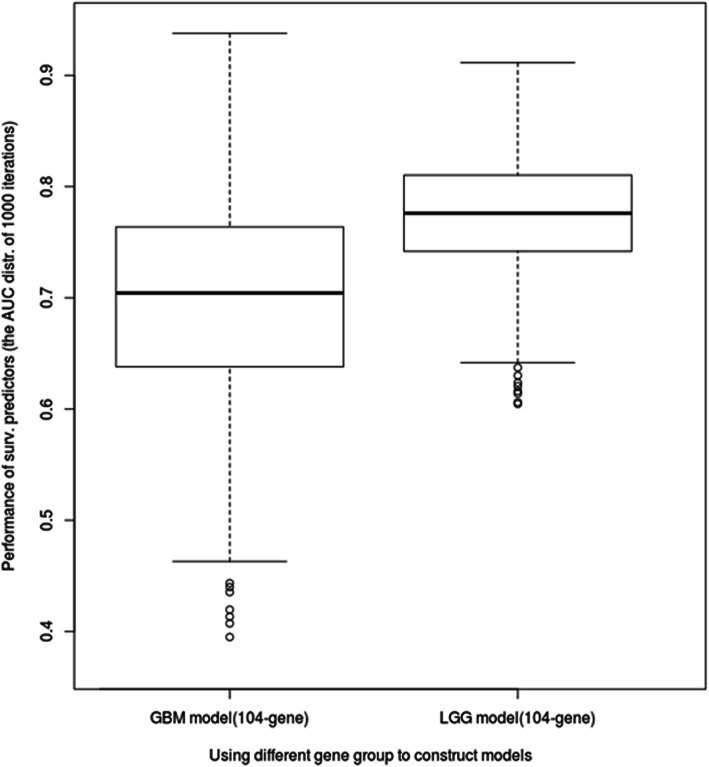
Performance estimation of survival prediction models using the 104-gene group (TCGA samples). The X-axis represents two survival prediction models that were constructed with the 104 survival-related genes; one model was constructed for GBM and the other, for LGG. The Y-axis represents the distribution of AUC values after 1000 repetitions of 5-fold cross-validation

In order to estimate the effectiveness of the 104 shared survival-related genes, two survival prediction models were constructed with 1000 iterations of five-fold cross-validation for the GBM and LGG cohorts. The area under the curve (AUC) value distribution under the 1000-time simulation was illustrated using the boxplot and summarized (Fig. 3; Table 3). The mean AUC values of the GBM and LGG models were estimated approximately from 0.7 to 0.8 and the standard deviations were from 0.05 to 0.09. Therefore, it was seen that the 104 genes affected the survival durations of patients with GBM and LGG to a certain extent.

**Table 3:** Capability estimation of 104 key genes for GBM and LGG survival prediction

Type	GBM	LGG
Minimum	0.40	0.60
Maximum	0.94	0.91
Mean	0.70	0.77
Standard deviation	0.09	0.05

### Pathway involvement and function category of survival-related genes

The 104 genes identified were common regulators related to the survival of GBM and LGG and were further analyzed for their involvement in pathways and possible biological roles. The results overlapped in at least two of the three tools; eight pathways were identified as core pathways (Table 4). Half of these pathways were signal transduction pathways correlated with cell survival, death, and growth. The molecular and cellular functions of the 104-gene group could be characterized using 23 biological functions (Table 5). In addition, IPA analysis revealed that these genes were able to be correlated to several mechanism disorders such as those related to immunity, inflammation, tissue connectivity, cellular movement, immune cell trafficking, cell death and survival, and cell-to-cell signaling and interaction.

**Table 4:** Pathways summarized from the enrichment analysis of the 104 survival-related genes

Pathway	GeneAnalytics	IPA	DAVID
ERK signaling	✓	✓	
Integrin pathway	✓	✓	
Akt signaling	✓	✓	
Phospholipase-C pathway	✓	✓	
Inhibition of matrix metalloproteases		✓	✓
TNF signaling pathway	✓		✓
Hematopoietic cell lineage	✓		✓
Jak-STAT signaling pathway		✓	✓

**Table 5:** Molecular and cellular functions summarized from the enrichment analysis of the 104 survival-related genes

Molecular and cellular function	GeneAnalytics	IPA	DAVID
Endodermal cell differentiation	✓		✓
Chemotaxis	✓		✓
T-cell activation	✓		✓
Extracellular matrix organization	✓		✓
Decidualization	✓		✓
Cytokine-mediated signaling pathway	✓	✓	✓
Heterotypic cell–cell adhesion	✓		✓
Angiogenesis	✓		✓
Adaptive immune response	✓		✓
Integrin-mediated signaling pathway	✓	✓	✓
Positive regulation of cell-substrate adhesion	✓		✓
Substrate-adhesion-dependent cell spreading	✓		✓
Positive regulation of tyrosine phosphorylation of Stat3 protein	✓		✓
Collagen fibril organization	✓		✓
Negative regulation of JAK-STAT cascade	✓		✓
Spongiotrophoblast differentiation	✓		✓
Leukocyte migration	✓		✓
Skeletal system development	✓	✓	✓
T-cell migration	✓		✓
Positive regulation of T cell proliferation	✓	✓	✓
Regulation of vesicle-mediated transport	✓		✓
positive regulation of T cell chemotaxis	✓		✓
Movement of cell or subcellular component		✓	✓

### The candidate patients’ severity-relevant features

**Fig. 4:**
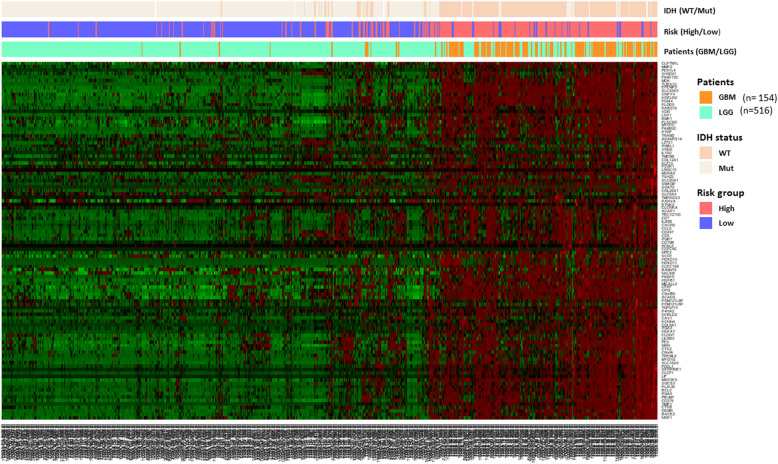
Heatmap view of the unsupervised clustering of 670 patients with glioma with expression profiles of the 104-gene group (TCGA samples). In the heatmap, the Y-axis represents the 104 genes and the X-axis represents patients with glioma. The expression levels from low to high are represented as a color gradient from green to red, respectively. There are three color bars of the heatmap utilizes different colors to represent IDH status (wild type and mutation), risk group (high/low), and patients with LGG and GBM

Unsupervised clustering with the 104 survival-related genes in all glioma patients (GBM and LGG) revealed that the expression levels of these genes would be higher in most of GBM cases and lower in LGG cases. This property could be applied as an indicator to distinguish patients’ risk (Fig. 4). Expression level analysis of the 104 shared genes relative to the expression of the 5 control genes were conducted among all patients with LGG (*n* = 516) and GBM (*n* = 154) from TCGA. Eighty-six of these genes, however, were screened between the different study cohorts for their signatures; subsequently, the other genes would be skipped here because they could not be validated with different datasets from GEO. For each feature, the selection criteria applicable state that more than 50 % of patients with GBM and LGG must have a different expression tendency relative to that of the control genes. Consequently, 19 features (with 16 genes involved) that met these criteria were filtered and then validated using various microarray datasets (Table 6). Obviously, two control genes, *GAPDH* and *RPL13A,* were filtered out in this study, because the expression levels of survival-related genes relative to both these control genes did not have clear differences in case of GBM and LGG. Additionally, 16 genes involved in these features had a higher expression in GBM than in LGG.

**Table 6:** Candidate features have different expression level between GBM and LGG

	TCGA		GSE16011		GSE4271		GSE4412	
	GBM	LGG	GBM	LGG	GBM	LGG	GBM	LGG
*CTSZ/IPO8*	+ (79.22%)	- (65.12%)					+ (62.71%)	- (80.77%)
*EFEMP2/IPO8*	+ (78.57%)	- (89.15%)	+ (83.02%)	- (64.22%)				
*EFEMP2/SDHA*	+ (58.44%)	- (96.12%)	+ (52.83%)	- (85.32%)	+ (61.84%)	- (83.33%)		
*EFEMP2/TBP*	+ (94.16%)	- (50.39%)	+ (81.76%)	- (64.22%)			+ (79.66%)	- (65.38%)
*ITGA3/IPO8*	+ (52.60%)	- (88.37%)					+ (62.71%)	- (92.31%)
*ITGA5/IPO8*	+ (56.49%)	- (95.54%)	+ (60.38%)	- (79.82%)				
*KDELR2/SDHA*	+ (98.70%)	- (70.74%)	+ (83.02%)	- (64.22%)				
*LITAF/SDHA*	+ (80.52%)	- (79.46%)	+ (88.68%)	- (54.13%)				
*MAP 2 K3/TBP*	+ (90.26%)	- (63.37%)					+ (69.49%)	- (61.54%)
*MDK/IPO8*	+ (85.06%)	- (87.02%)	+ (76.73%)	- (74.31%)			+ (79.66%)	- (73.08%)
*MICALL2/TBP*	+ (83.77%)	- (53.10%)	+ (81.76%)	- (55.96%)				
*NRP1/IPO8*	+ (57.79%)	- (92.83%)	+ (57.86%)	- (73.39%)	+ (86.84%)	- (70.83%)		
*P4HA2/TBP*	+ (57.79%)	- (85.27%)	+ (62.89%)	- (86.24%)				
*PDIA4/SDHA*	+ (94.81%)	- (75.78%)			+ (78.95%)	- (70.83%)		
*PLAUR/TBP*	+ (71.43%)	- (89.73%)					+ (67.80%)	- (92.31%)
*PLOD3/SDHA*	+ (50.09%)	- (92.25%)	+ (84.91%)	- (55.96%)				
*SERPINE1/IPO8*	+ (68.18%)	- (88.76%)	+ (67.30%)	- (77.98%)	+ (76.32%)	- (70.83%)	+ (84.75%)	- (65.38%)
*SERPINE1/TBP*	+ (96.10%)	- (60.08%)	+ (69.18%)	- (79.82%)				
*SOCS3/IPO8*	+ (50.65%)	- (92.64%)					+ (74.58%)	- (69.23%)

The positive sign “+” means the expression level of survival-related gene comparing to the control gene (log2 ratio) was higher. The negative sign “-” means the expression level of survival-related gene comparing to the control gene (log2 ratio) was lower. The percentage represents how many patients in GBM or LGG are expressed higher/lower than the control genes

### Effectiveness features for evaluating risks of patients with glioma

Based on the assumption that patients with GBM (*n* = 154) have a higher risk (short median OS) than those with LGG (*n* = 516) (longer median OS), the construction of a logistic regression model with various combinations of features was repeated. The ten smallest features, namely, *CTSZ/IPO8, EFEMP2/IPO8*, *ITGA5/IPO8*, *KDELR2/SDHA*, *MDK/IPO8*, *MICALL2/TBP*, *MAP 2 K3/TBP, PLAUR/TBP*, *SERPINE1/TBP*, and *SOCS3/IPO8* were utilized to construct the risk model with the lowest AIC value which was 239.51. Therefore, utilizing the signatures of ten genes relative to the three control genes would have the capability to evaluate patient risks.

### Patients’ risk distinguishable with ten gene signatures

**Fig. 5:**
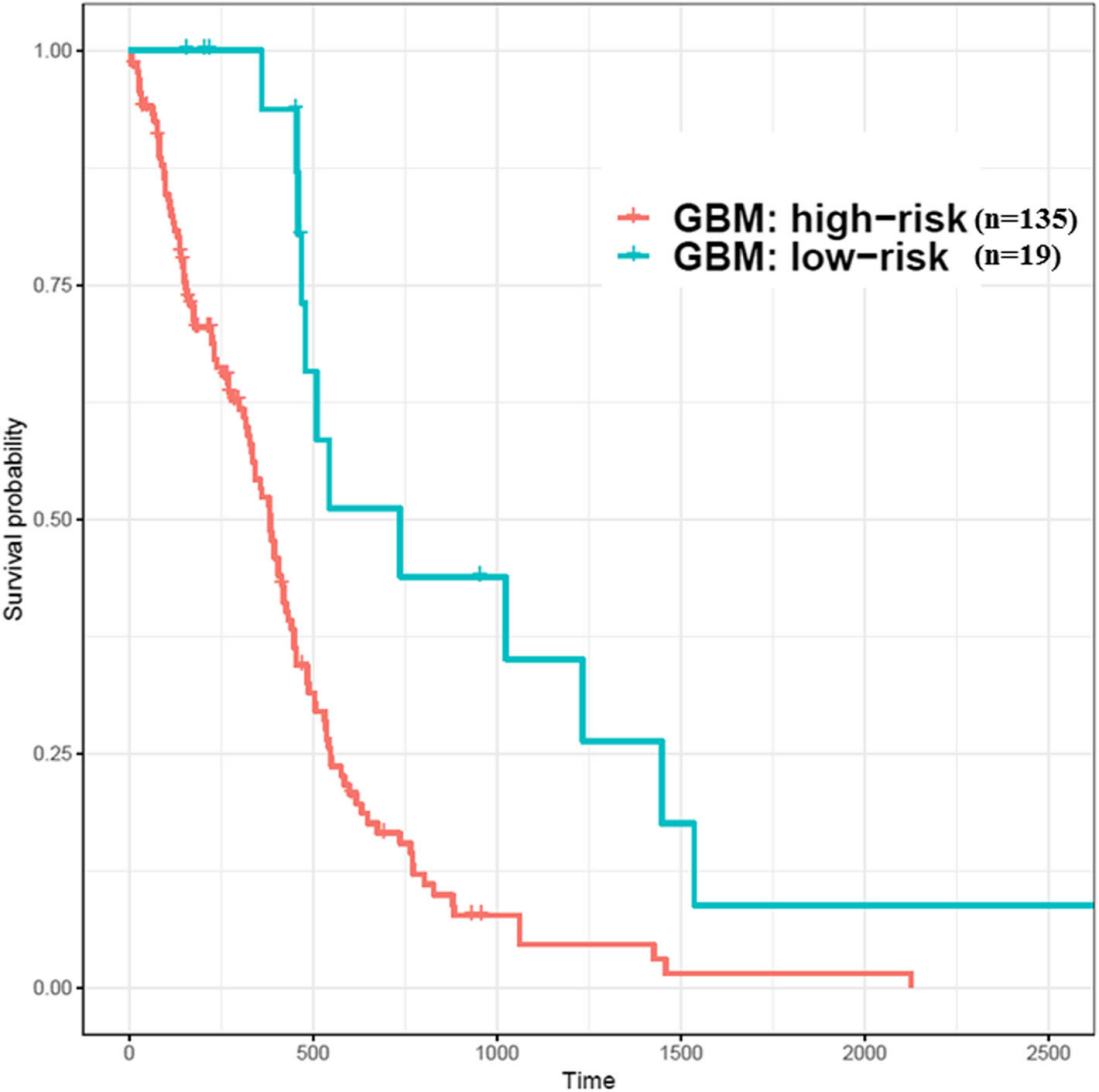
Patients with GBM were divided into high- and low-risk groups identified based on ten genes (TCGA samples). X-axis: patients’ OS duration (days); Y-axis: patients’ survival rate. Log rank test between high-risk (*n* = 135) and low-risk (*n* = 19) groups was significant difference (*p* < 0.01)

**Fig. 6:**
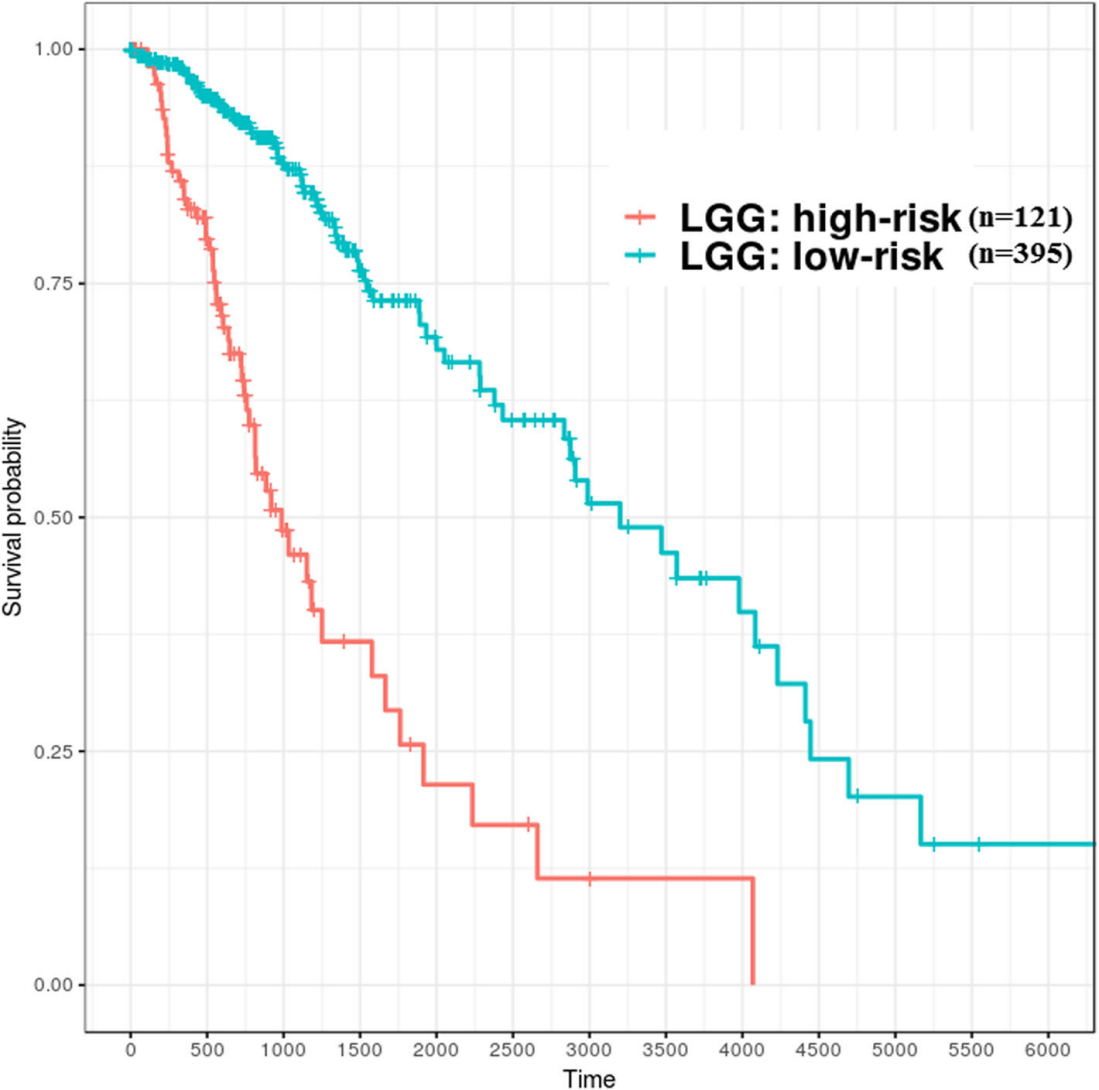
Patients with LGG were divided into high- and low-risk groups identified based on ten genes (TCGA samples). X-axis: patients’ OS duration (days); Y-axis: patients’ survival rate. Log rank test between high-risk (*n* = 121) and low-risk (*n* = 395) groups was significant difference (*p* < 0.01)

**Fig. 7:**
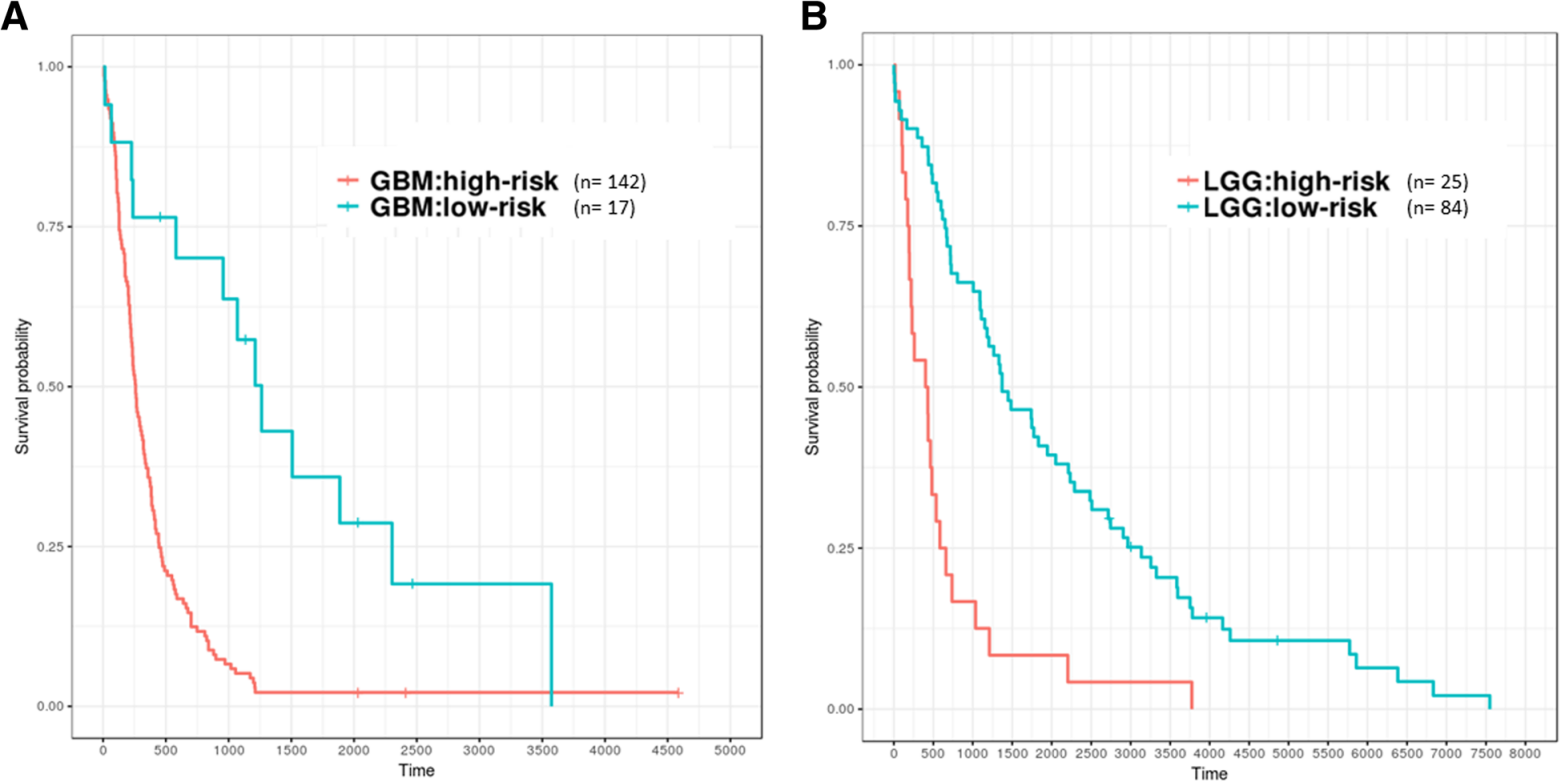
Patients with GBM and LGG were divided into high- and low-risk groups identified based on ten genes (GEO samples). X-axis: patients’ OS duration (days); Y-axis: patients’ survival rate. **a** GBM, log rank test between high-risk (*n* = 142) and low-risk (*n* = 17) groups was significant difference (*p* < 0.01). **b** LGG, log rank test between high-risk (*n* = 25) and low-risk (*n* = 84) groups was significant difference (*p* < 0.01)

After screening the importance of features with the logistic regression model, ten of these features would be used to construct the risk models for GBM and LGG. In the GBM study cohort, when the probability that patients belong to the high-risk group was less than 0.35, they would be clustered into the relatively low-risk group, whereas the LGG risk model attempted to identify relatively high-risk patients when this probability was larger than 0.9. Therefore, GBM patients (*n* = 154) could be divided into the high-risk group (*n* = 135) and low-risk group (*n* = 19); LGG patients (*n* = 516) could also be divided into the low-risk group (*n* = 364) and high-risk group (*n* = 152). The risk groups shown in the Kaplan-Meier curve (Fig. 5 and Fig. 6) were significantly different (Log rank test, *p* < 0.01). Moreover, the models were evaluated by repeating the 5-fold cross-validation 1000 times; the average AUC value of ROC in GBM was 0.986, and that in LGG was 0.982. In addition to testing effectiveness of candidate genes which were used to construct risk models against TCGA datasets (RNA-Seq), microarray dataset GSE16011 which include large cases (GBM, *n* = 159; LGG, *n* = 109) and with various grades (G2, G3, and G4) was used to validate it. However, datasets from different platforms, the distribution of overall gene expression level would be varies. Thus, building different risk models for GBM and LGG for various platforms is required. Consequently, the Kaplan-Meier curve showed that GBM and LGG from GSE16011 can be well distinguished into different risk groups (log rank test, *p* < 0.01) using the candidate features (Fig. 7).

## Discussion

In this study, we identified 104 common survival-related genes from patients with gliomas. The effectiveness of these genes was evaluated by constructing prediction models, and the AUC values were estimated to be approximately 0.7 and 0.8 for the GBM and LGG models, respectively, after 1000 iterations of 5-fold cross-validation. The heatmap (Fig. 4) has shown that expression profiles of these genes are associated with the *IDH1* and risk status among patients with gliomas; most of patients with GBM are wild-type *IDH1* and have short survival time (high risk), but patients with LGG are mutant-type *IDH1* and survive long (low risk). Most of these genes were involved in cell-related signaling pathways that affect cellular proliferation, apoptosis, and angiogenesis. Moreover, of the 104 survival-related genes, 10 could potentially distinguish patients with GBM or LGG into high- and low-risk groups. The expression levels of these ten genes were higher and the survival duration was shorter in patients with high-grade glioma than in those with lower grade glioma.

Identification of survival-related genes in gliomas has been ongoing over the past decade. However, the gene lists identified by our study and the other various research groups [14–29] differ considerably; only 13 common genes (*BMP2, CLIC1, EST, IGFBP2, LDHA, LGALS1, MET, MSN, TGALN2, TIMP1, TNC, UPP1, ZYX*) could be identified in at least three studies. These differences may be attributed to two major factors. First, researchers have analyzed glioma datasets from various perspectives; for instance, some studies have discussed some aspects only in patients with high-grade gliomas [14, 20, 23–27, 29], or LGG [21, 28], or at specific checkpoints such as the mitotic spindle checkpoint [19] or ion channel [27]. Second, studies have analyzed different types of datasets obtained from various high-throughput platforms such as microarray or next-generation data. Because of technical limitations, the expression profiles of the same genes detected from different datasets may be inconsistent. For instance, the sequencing data have higher stochastic variability than array data, which would result in a lack of reads in short or low abundance genes [40]. On the other hand, microarray data of gene expression can be affected by probes’ cross-hybridization, nonspecific hybridization, redundancy, and annotation [41]. Rather than NGS, microarray analysis has been selected as the initial screening method in most relevant studies. Recently, various research groups have started using NGS data (e.g., RNA-Seq) as the main analysis platform and microarray data as an adjuvant platform to verify results.

Accurate survival prediction through comprehensive indicators is vital for patients with glioma. However, the 104-gene group identified in this study would include parts of those indicators and was also crucial for survival prediction in both types of gliomas; its effectiveness in analyzing the GBM and LGG cohorts demonstrated that there other specific survival-related genes might exist. However, these 104 genes were the basic factors for patients’ survival in case of GBM and LGG, because the average AUC value under multiple times of simulation could reach 0.7–0.8. These genes could be used together with self-specific genes of each type of glioma, to elaborate the regulation networks in various mechanisms. In addition, recent studies have identified crucial glioma imaging features from magnetic resonance imaging (MRI) and have correlated them with patient survival [42, 43]. The association of imaging and genomic features could be realized and applied in the field of radiogenomics.

The expression level analysis of survival-related genes could have implications; high signatures of genes in patients would be indicative of shorter survival durations in contrast to low signatures of genes, where patients have a longer survival time. Moreover, most of these genes were highly expressed in GBM and the converse is true in case of LGG. However, it is difficult to set the cutoff values to indicate whether gene expression was high or low, because of individual differences. Therefore, the reference genes (called control genes in this study) would be the target of comparison for survival-relevant genes. Furthermore, in order to have objective indicators consequently, different datasets were used to validate the results from NGS and the minor effectiveness of genes, which was decided by the logistic regression model, was removed.

**Fig. 8:**
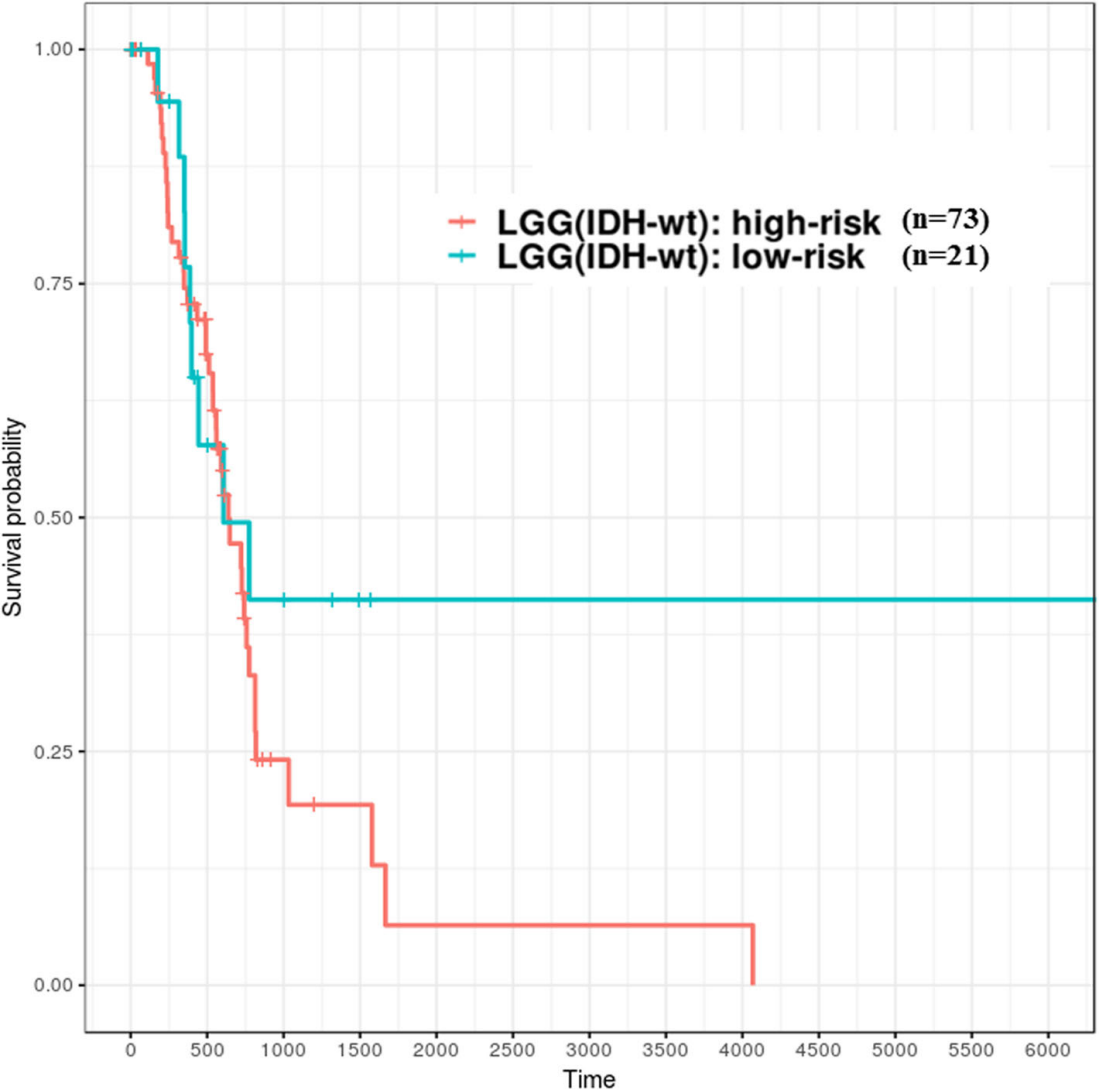
High- and low-risk groups of patients with LGG having wild-type *IDH1* identified based on ten genes (TCGA samples). X-axis: patients’ OS duration (days); Y-axis: patients’ survival rate. Both risk groups could not be separated well; log rank test, *p* = 0.248 was not statistically significant

**Fig. 9:**
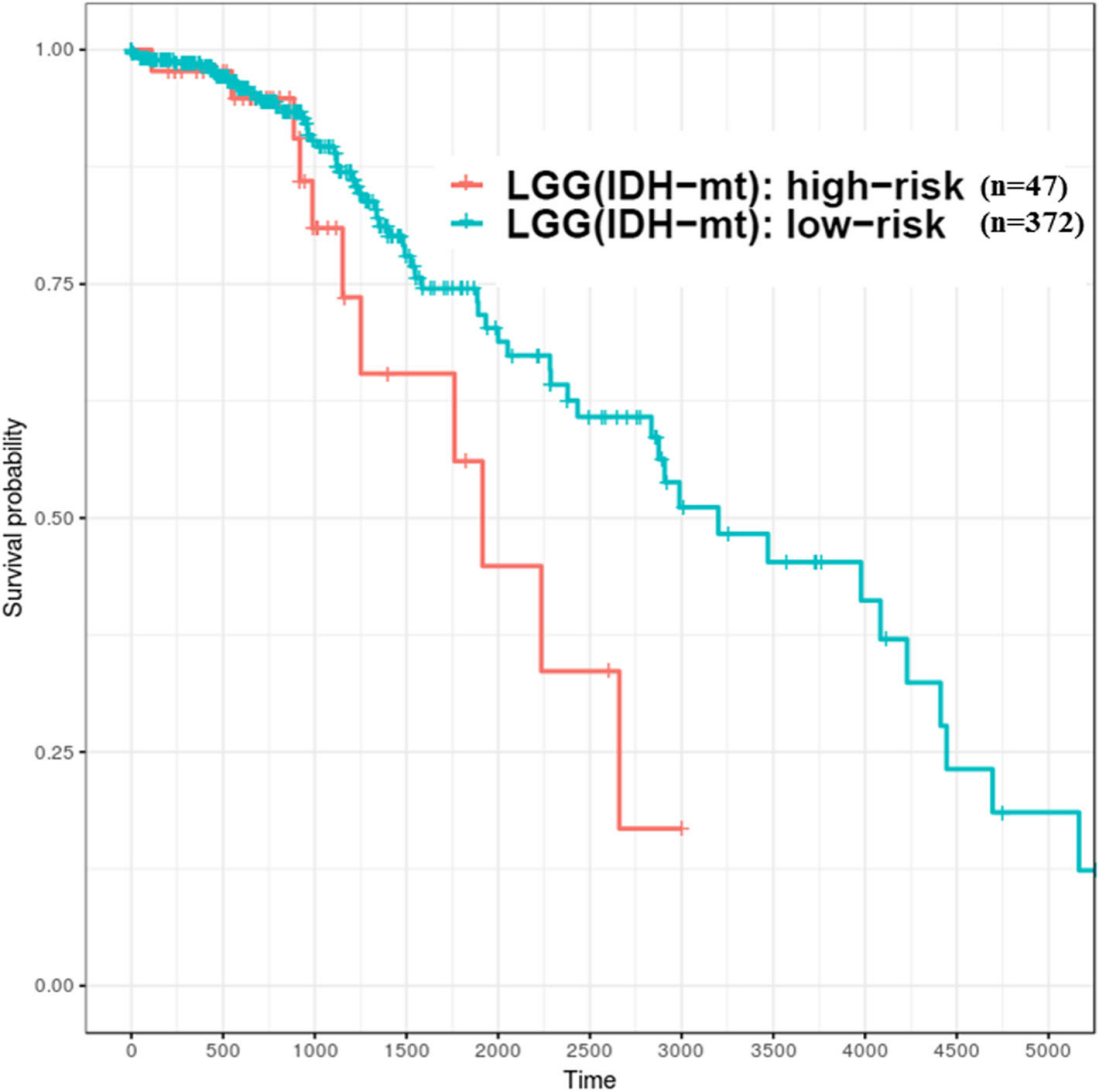
High- and low-risk groups of patients with LGG having mutant-type *IDH1* identified based on ten genes (TCGA samples). X-axis: patients’ OS duration (days); Y-axis: patients’ survival rate. Log rank test between high-risk (*n* = 47) and low-risk (*n* = 372) groups was significant difference (*p* < 0.05)

Survival-relevant risk models were constructed for GBM and LGG; the evaluation related to model performance was larger than 0.95 (average AUC value), which means it could successfully classify patients into different risk groups. In GBM, the survival durations of the low-risk group were better than the high-risk group, and its median OS was larger than 450 days (1.2 years). On the other hand, in LGG, the survival durations of the high-risk group were worse than that in the low-risk group and the median OS time was shorter than 2700 days (7.4 years). Recent studies [44–47] have demonstrated that gliomas could be divided into multiple subtypes based on various molecular features such as *IDH1* mutation/wild type and chromosome 1p/19q noncodeletion/codeletion. Generally, *IDH1* mutation and 1p/19q codeletion are favorable prognostic factors for patients with gliomas. Patients with GBM (*n* = 154) from TCGA, in addition to the following status: chromosome 1p/19q was not available (NA), all showed noncodeletion; only 10 patients showed *IDH1* mutation. In the low-risk group (*n* = 19) of GBM, as identified using the logistic regression model, 8 patients had the *IDH1* mutant. This result indicated that 80% of patients with *IDH1* mutation would be clustered into the relative low-risk group of GBM against the risk prediction model. Unlike GBM, *IDH1* was mutated in most patients (*n* = 419) and fewer patients (*n* = 94) had the wild-type *IDH1* in the LGG (*n* = 516) datasets from TCGA. In addition, with regards to the status observation of chromosome 1p/19q for patients with LGG, parts (*n* = 347) of them were noncodeletion and the others (*n* = 169) were codeletion. According to the relative high-risk group (*n* = 121) of LGG clustered by our constructed model, most of patients had wild-type *IDH1* (*n* = 73) and 1p/19q-noncodeletion (*n* = 108), accounting for 60.3 and 89.3% of the cases, respectively (Table 7). Therefore, the molecular features of the high- and low-risk groups identified using the signatures of these ten genes were in accordance with the results of previous studies. However, the risk model could not directly classify patients with wild-type *IDH1* into different risk groups well. For instance, 22.3% (*n* = 21) and 77.7% (*n* = 73) of patients with wild-type *IDH1* in the LGG cohort (*n* = 94) were identified as low and high risk, respectively (Table 7); the survival curves of both risk groups could not be separated significantly, especially those representing less than 750 days of survival durations (Fig. 8). Therefore, further identification of other effective predictors is required to evaluate how patients with better survival can be efficiently distinguished from patients with glioma having wild-type *IDH1*. However, the risk model could classify patients with mutant-type *IDH1* into different risk groups well in LGG cohort (*n* = 419). There are 47 (11.2%) and 372 (88.8%) of patients belong to high- and low-risk groups, respectively (Fig. 9). The high-risk group of patients with *IDH1* mutation might be associated with DNA methylation, which has indicated in the study based on the molecular profiling analysis [48].

**Table 7:** *IDH1* and 1p/19q status of high- and low-risk groups of patients with LGG and those with GBM

	LGG		GBM	
	High risk	Low risk	High risk	Low risk
n	121	395	135	19
*IDH1* status				
Wild type	73 (60.3%)	21 (5.3%)	130 (96.3%)	10 (52.6%)
Mutation	47 (38.8%)	372 (94.2%)	2 (1.5%)	8 (42.1%)
Unknown	1 (0.8%)	2 (0.5%)	3 (2.2%)	1(5.3%)
1p/19q status				
Codel	13 (10.7%)	156 (39.5%)		
Noncodel	108 (89.3%)	239 (60.5%)	130 (96.3%)	18 (94.7%)
Unknown			5 (3.7%)	1 (5.3%)
*IDH1* and 1p/19q status				
Wild type and noncodel	73 (60.3%)	21 (5.3%)	126 (93.3%)	10 (52.6%)
Mutation and noncodel	34 (28.1%)	216 (54.7%)	1 (0.7%)	7 (36.8%)
Mutation and codel	13 (10.7%)	156 (39.5%)		
Wild type			4 (3.0%)	
Mutation			1 (0.7%)	1 (5.3%)
Codel				
Noncodel	1 (0.8%)	2 (0.5%)	3 (2.2%)	1 (5.3%)

**Fig. 10:**
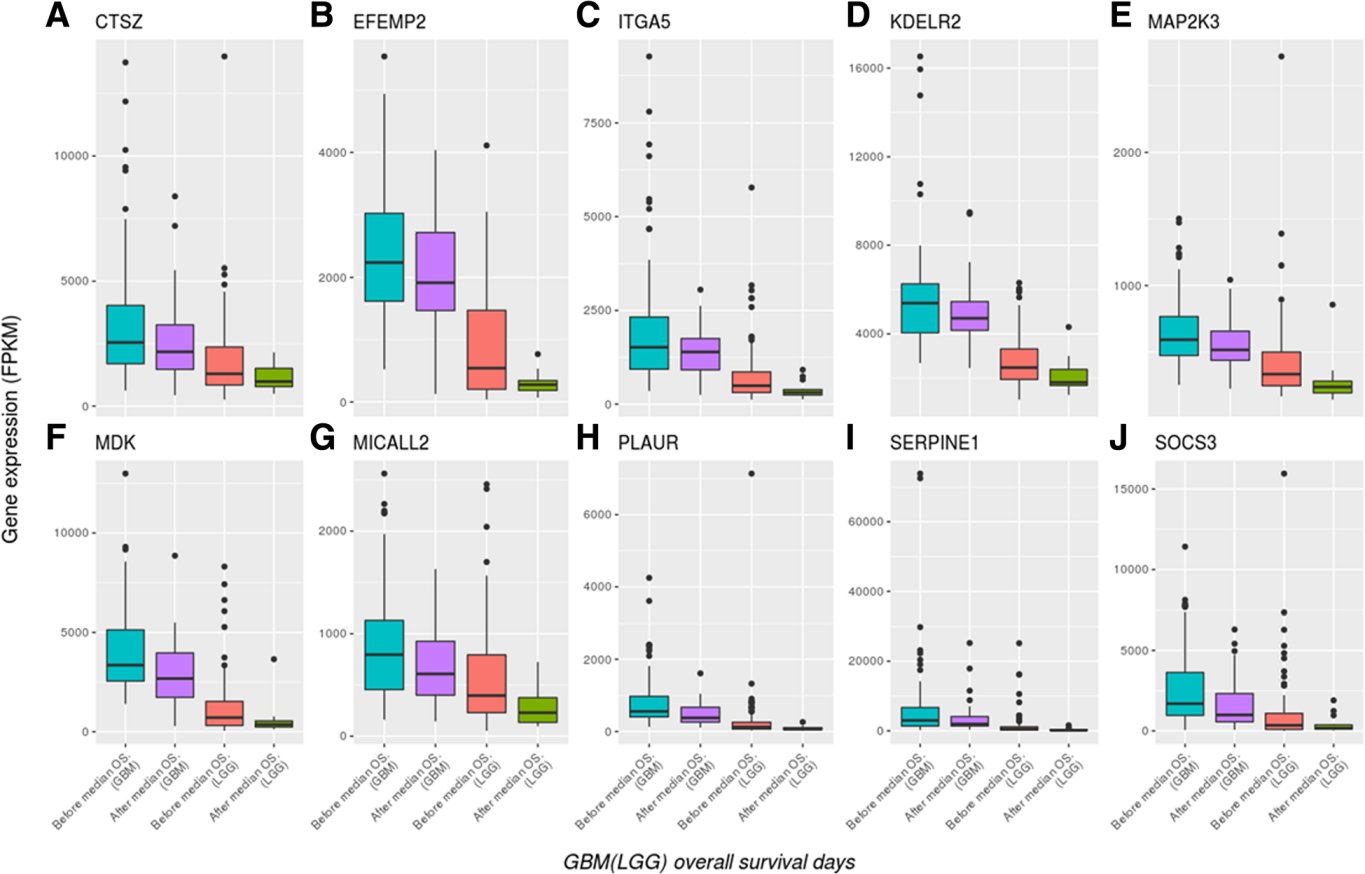
Expression levels of ten genes decreased from GBM to LGGs (TCGA samples). All patients with glioma (*n* = 247) were dead (121 patients with GBM and 126 patients with LGG). The X-axis represents the patients’ OS duration and the Y-axis represents their gene expression levels (FPKM). The X-axis labels from left to right are: “Before median overall survival (OS) of GBM,” “After median OS of GBM,” “Before median OS of LGG,” and “After median OS of LGG”

The expression level of the ten aforementioned genes tended to gradually decrease from GBM to LGGs (Fig. 10). The OS duration of patients decreased upon high gene expression but increased upon low gene expression. Several of these genes have been reported in previous studies; for instance, *EFEMP2* was indicated as a potent oncogene in glioma and a target for glioma treatment [49], the overexpression of *SERPINE1* (*PAI-1*) was significantly correlated with shorter survival durations in patients with GBM [50], the expression of *ITGA5* might be correlated to the regulation of cell proliferation and invasiveness in GBM, because targeting *ITGA5* using miR-330-5p could affect these cell events [51], and the biological function of *PLAUR* could be related to glioma cell invasion and angiogenesis [52]. In addition, the ten genes could be mapped to the various hallmarks of cancer with literature survey (Table 8). Therefore, this 10-gene group might have potential prognostic value for patients with glioma.

**Table 8:** The cancer hallmarks mapping of the ten genes

Gene symbol	MSigDB (Hallmark) [53]	Hallmarks of cancer	Ref.
*CTSZ*	IL2/JAK/STAT5 signaling	sustaining proliferative signaling, activating invasion and metastasis	[54]
*EFEMP2*	Epithelial mesenchymal transition	sustaining proliferative signaling, activating invasion and metastasis	[49]
*ITGA5*	Epithelial mesenchymal transition, Inflammatory response	All	[55]
*KDELR2*		activating invasion and metastasis	[56]
*MDK*	Estrogen response late, Apical junction	sustaining proliferative signaling, activating invasion and metastasis	[57]
*MICALL2*		sustaining proliferative signaling	[58]
*MAP 2 K3*	TNF-a signaling via NF-kB, PI3K/AKT/mTOR signaling, mTORC1 signaling, Heme metabolism	sustaining proliferative signaling	[59]
*PLAUR*	TNF-alpha signaling via NF-kB, Cholesterol homeostasis	activating invasion and metastasis, inducing angiogenesis	[52, 60–62]
*SERPINE1*	TNF- alpha signaling via NF-kB, Hypoxia, TGF-beta signaling, Complement, Epithelial mesenchymal transition, Inflammatory response, Xenobiotic metabolism, UV response down, Coagulation	inducing angiogenesis	[63]
*SOCS3*	TNF- alpha signaling via NF-kB, IL6/JAK/STAT3 signaling, Interferon gamma response	sustaining proliferative signaling, tumor-promoting inflammation	[64]

## Conclusions

In summary, the 104 genes identified, which are common between patients with GBM and those with LGG, can be used as core genes related to patient survival. Of these, 10 genes (CTSZ, *EFEMP2*, *ITGA5*, *KDELR2*, *MDK*, *MICALL2*, *MAP 2 K3, PLAUR*, *SERPINE1*, and *SOCS3*) can potentially serve as indicators to classify patients with gliomas into different risk groups and could be used to estimate the prognosis of patients with gliomas. Moreover, the expression profiles of these potential biomarkers could be correlated to the molecular subtypes of patients, such as *IDH1/2* mutation/wild type and chromosome 1p/19q codeletion/noncodeletion.

## Additional file

Additional file 1: **Table S1.** Title: 104 common survival-related genes were identified from patients with GBM and those with LGG. Description: Summarization of Cox model results for 104 survival-relevant common genes between LGG and GBM with table. (PDF 38 kb)
