# Supplementary material for: Identification of potential biomarkers related to glioma survival by gene expression profile analysis
Source: BMC Med Genomics. 2019 Mar 20;11(Suppl 7):34. doi: 10.1186/s12920-019-0479-6 (PMC7402580; doi:10.1186/s12920-019-0479-6)
Supplement: Supplementary file 1 — Table S1. Title: 104 common survival-related genes were identified from patients with GBM and those with LGG. Description: Summarization of Cox model results for 104 survival-relevant common genes between LGG and GBM with table. (PDF 38 kb) [file 12920_2019_479_MOESM1_ESM.pdf]

Table S1. 104 common survival-related genes were identified from patients with GBM and those with LGG.

Gene symbol	LGG			GBM		
	HR (95% CI for HR)	Wald test, p value	Before and after median OS (t test, p value)	HR (95% CI for HR)	Wald test, p value	Before and after median OS (t test, p value)
**ACADS**	1.4 (1.2-1.6)	4.50E-06	2.49E-04	1.3 (1.1-1.6)	1.50E-03	4.77E-02
**ACAP1**	1.6 (1.4-1.8)	2.50E-14	5.20E-05	1.3 (1.1-1.5)	1.20E-03	1.68E-02
**ADAMTS14**	1.2 (1.1-1.3)	7.50E-04	4.32E-03	1.2 (1.1-1.4)	6.00E-03	9.87E-04
**B3GNT9**	1.5 (1.2-1.7)	1.20E-05	2.20E-02	1.3 (1.1-1.5)	4.40E-03	3.10E-02
**BACE2**	1.5 (1.3-1.6)	3.30E-14	6.96E-04	1.4 (1.1-1.6)	6.80E-04	2.40E-03
**BCL3**	1.4 (1.3-1.6)	2.80E-10	7.91E-03	1.2 (1.1-1.4)	7.20E-03	2.86E-02
**BMP1**	1.6 (1.4-1.9)	4.00E-13	3.71E-02	1.3 (1.1-1.5)	3.10E-03	8.48E-03
**C8orf4**	1.2 (1.1-1.4)	1.30E-03	1.52E-03	1.3 (1.1-1.6)	9.80E-03	1.92E-02
**C9orf89**	1.5 (1.3-1.7)	4.50E-07	1.98E-02	1.3 (1.1-1.5)	7.10E-03	4.67E-02
**CAV1**	1.2 (1.1-1.4)	7.50E-03	8.64E-03	1.3 (1.1-1.5)	4.20E-03	1.31E-03
**CCL5**	1.4 (1.3-1.6)	5.00E-09	5.00E-06	1.2 (1.1-1.5)	8.30E-03	3.86E-02
**CD247**	1.5 (1.3-1.7)	3.10E-09	9.30E-05	1.2 (1.1-1.5)	8.40E-03	2.69E-02
**CD276**	1.6 (1.4-1.8)	1.10E-13	1.03E-03	1.3 (1.1-1.5)	2.00E-03	1.17E-04
**CD6**	1.4 (1.2-1.6)	1.80E-06	7.65E-03	1.3 (1.1-1.5)	6.80E-03	4.21E-02
**CD7**	1.2 (1.1-1.3)	3.30E-03	1.74E-03	1.3 (1.1-1.5)	4.80E-03	4.53E-02
**CD79B**	1.7 (1.4-2.1)	5.80E-07	1.90E-03	1.2 (1.1-1.4)	4.90E-03	3.39E-02
**CLCF1**	1.3 (1.2-1.4)	8.40E-09	6.58E-04	1.3 (1.1-1.6)	1.40E-03	2.23E-02
**CLCNKA**	1.2 (1.1-1.3)	3.90E-06	7.31E-04	1.2 (1.1-1.5)	6.70E-03	4.60E-02
**CLDN7**	1.3 (1.1-1.5)	1.40E-04	2.51E-02	1.4 (1.1-1.7)	4.90E-03	7.93E-03
**CLEC18A**	1.5 (1.3-1.6)	1.10E-13	0.00E+00	1.3 (1.1-1.6)	3.50E-03	3.25E-02
**CLEC4C**	1.2 (1.1-1.4)	6.90E-04	2.62E-03	1.3 (1.1-1.6)	9.20E-04	4.58E-03
**CLPTM1L**	1.3 (1.1-1.5)	3.20E-04	6.35E-04	1.3 (1.1-1.5)	4.90E-04	8.36E-03
**CNPY4**	1.8 (1.5-2.1)	5.30E-15	4.41E-03	1.4 (1.2-1.6)	9.10E-05	4.32E-02
**COL12A1**	1.4 (1.3-1.6)	3.30E-11	3.43E-02	1.4 (1.1-1.8)	2.50E-03	3.61E-02
**COL22A1**	1.4 (1.3-1.6)	1.10E-09	3.87E-04	1.4 (1.2-1.7)	3.70E-05	4.17E-03
**COL8A1**	1.3 (1.2-1.4)	5.00E-15	2.10E-04	1.3 (1.1-1.5)	6.40E-03	2.56E-02
**CPQ**	2.1 (1.8-2.5)	0.00E+00	3.00E-06	1.4 (1.1-1.7)	5.40E-04	4.71E-02
**CTSB**	1.6 (1.4-1.8)	2.10E-10	2.04E-02	1.4 (1.1-1.6)	9.60E-04	4.24E-03

**CTSZ**	1.5 (1.3-1.7)	1.70E-08	5.63E-03	1.3 (1.1-1.6)	6.10E-03	4.36E-02
**CXCR6**	1.5 (1.3-1.8)	1.30E-07	2.00E-06	1.3 (1.1-1.6)	3.30E-03	3.98E-02
**DCBLD2**	1.3 (1.2-1.4)	4.80E-09	2.15E-04	1.2 (1.1-1.5)	5.00E-03	1.13E-02
**EFEMP2**	2 (1.8-2.3)	0.00E+00	0.00E+00	1.5 (1.2-1.8)	9.30E-05	1.15E-02
**ETNK2**	1.2 (1.1-1.3)	3.00E-05	2.79E-02	1.2 (1.1-1.5)	9.90E-03	3.34E-02
**FABP5**	1.5 (1.3-1.6)	0.00E+00	1.00E-06	1.3 (1.1-1.6)	6.90E-03	2.99E-02
**FAM115C**	1.3 (1.2-1.5)	3.90E-06	1.86E-03	1.3 (1.1-1.6)	1.50E-03	1.37E-02
**FAM20C**	1.4 (1.3-1.5)	1.30E-10	2.26E-03	1.4 (1.2-1.6)	3.50E-04	3.13E-03
**FBLIM1**	1.2 (1.1-1.4)	3.90E-04	2.22E-03	1.2 (1.1-1.5)	8.40E-03	6.72E-03
**FCRL5**	1.6 (1.3-2)	9.70E-05	4.90E-02	1.4 (1.2-1.7)	9.50E-05	6.51E-03
**FER1L4**	1.2 (1.1-1.3)	8.70E-05	2.98E-02	1.3 (1.1-1.6)	1.60E-03	2.69E-02
**FES**	1.5 (1.3-1.8)	6.00E-10	1.12E-03	1.3 (1.1-1.6)	1.90E-03	3.33E-02
**FOSL1**	1.2 (1.1-1.3)	8.00E-04	6.01E-03	1.4 (1.2-1.6)	1.80E-04	2.79E-02
**GRN**	1.5 (1.3-1.7)	7.60E-08	5.68E-04	1.3 (1.1-1.5)	9.70E-03	2.35E-02
**HOXD10**	1.3 (1.2-1.5)	6.80E-11	2.10E-05	1.3 (1.1-1.5)	5.90E-03	5.97E-03
**HOXD11**	1.4 (1.3-1.5)	1.90E-12	3.10E-05	1.3 (1.1-1.6)	4.80E-03	4.00E-02
**HSPA7**	1.2 (1.1-1.3)	6.90E-06	4.24E-04	1.3 (1.1-1.5)	4.50E-03	4.57E-02
**HSPB1**	1.6 (1.4-1.8)	1.60E-14	0.00E+00	1.3 (1.1-1.6)	1.70E-03	1.76E-02
**IL1R2**	1.2 (1.1-1.3)	8.70E-04	2.46E-02	1.3 (1.1-1.6)	5.00E-03	1.11E-02
**IL2RB**	1.3 (1.1-1.5)	6.80E-04	6.20E-05	1.3 (1.1-1.5)	5.20E-03	2.29E-02
**ITGA3**	1.4 (1.3-1.6)	3.40E-14	5.95E-04	1.3 (1.1-1.5)	1.90E-03	2.48E-02
**ITGA5**	1.2 (1.1-1.3)	4.40E-05	2.13E-04	1.3 (1.1-1.6)	5.10E-04	1.58E-03
**ITGB7**	1.2 (1.1-1.3)	1.20E-04	3.11E-04	1.3 (1.1-1.5)	1.20E-03	3.88E-02
**ITGBL1**	1.2 (1.1-1.3)	1.50E-03	1.11E-02	1.3 (1.1-1.5)	4.10E-03	4.24E-02
**KCNN4**	1.4 (1.2-1.6)	4.40E-07	2.20E-05	1.3 (1.1-1.5)	2.50E-03	1.03E-02
**KDELR2**	1.4 (1.3-1.6)	2.20E-12	5.03E-03	1.3 (1.1-1.5)	3.10E-03	4.12E-02
**KLF10**	1.4 (1.2-1.6)	1.10E-08	2.73E-04	1.3 (1.1-1.6)	2.90E-03	6.52E-03
**LIF**	1.2 (1.1-1.4)	1.10E-05	1.54E-02	1.3 (1.1-1.6)	2.20E-03	2.15E-02
**LILRB3**	1.5 (1.3-1.8)	1.30E-10	1.60E-05	1.3 (1.1-1.6)	3.60E-04	1.27E-03
**LITAF**	1.5 (1.3-1.7)	1.20E-07	3.34E-02	1.3 (1.1-1.6)	1.70E-03	9.96E-03
**LRRC15**	1.2 (1.1-1.4)	2.30E-03	7.35E-03	1.2 (1.1-1.5)	8.90E-03	2.17E-02
**LSP1**	1.7 (1.5-1.9)	0.00E+00	3.07E-03	1.3 (1.1-1.5)	5.80E-04	9.94E-03
**LZTS1**	1.4 (1.2-1.6)	9.60E-07	3.85E-02	1.2 (1.1-1.4)	9.40E-03	3.59E-03

**MAP2K3**	1.6 (1.4-1.8)	1.30E-15	1.11E-02	1.3 (1.1-1.6)	1.90E-03	1.25E-02
**MDK**	1.3 (1.2-1.5)	4.00E-07	2.78E-02	1.3 (1.1-1.5)	5.10E-03	4.15E-03
**MICALL2**	1.5 (1.3-1.6)	1.80E-11	1.08E-04	1.3 (1.1-1.5)	3.60E-03	1.01E-02
**MMP2**	1.2 (1.1-1.3)	3.60E-04	3.09E-03	1.4 (1.2-1.8)	4.40E-04	2.19E-02
**MXRA8**	1.5 (1.3-1.7)	1.60E-09	2.31E-03	1.4 (1.2-1.7)	3.00E-05	9.88E-03
**MYO1C**	1.3 (1.1-1.4)	1.60E-05	5.55E-03	1.3 (1.1-1.5)	6.40E-03	1.14E-02
**MYO1G**	1.3 (1.1-1.4)	3.90E-05	1.58E-02	1.3 (1.1-1.6)	3.10E-03	2.54E-02
**NFE2**	1.4 (1.2-1.6)	4.50E-07	2.38E-02	1.3 (1.1-1.6)	2.80E-03	6.67E-03
**NRP1**	1.4 (1.2-1.6)	3.60E-06	4.83E-02	1.3 (1.1-1.6)	3.40E-03	9.27E-04
**NSUN5**	1.3 (1.1-1.5)	2.10E-03	1.70E-02	1.4 (1.2-1.7)	3.10E-05	5.46E-03
**OSMR**	1.5 (1.4-1.7)	0.00E+00	9.00E-05	1.4 (1.2-1.7)	4.50E-04	1.64E-02
**P4HA2**	1.3 (1.2-1.5)	8.70E-06	1.22E-03	1.3 (1.1-1.5)	8.30E-04	2.13E-03
**PARVA**	1.2 (1.1-1.4)	8.30E-03	1.41E-02	1.4 (1.1-1.6)	1.50E-03	2.10E-02
**PDIA4**	1.7 (1.5-2)	0.00E+00	1.98E-02	1.3 (1.1-1.5)	4.40E-04	4.72E-03
**PDZK1**	1.4 (1.2-1.5)	6.40E-07	2.21E-03	1.3 (1.1-1.6)	5.70E-04	2.99E-02
**PLAUR**	1.3 (1.2-1.4)	2.90E-11	9.55E-03	1.4 (1.2-1.6)	1.10E-04	5.59E-04
**PLOD3**	1.7 (1.5-1.9)	6.70E-16	2.67E-04	1.2 (1.1-1.4)	6.10E-03	2.29E-02
**PLSCR3**	1.4 (1.2-1.6)	2.00E-05	6.20E-03	1.3 (1.1-1.6)	5.30E-03	7.11E-03
**POM121L8P**	1.3 (1.2-1.5)	1.30E-05	3.10E-03	1.4 (1.1-1.6)	4.20E-04	1.74E-02
**POM121L9P**	1.4 (1.2-1.5)	3.80E-06	4.10E-05	1.3 (1.1-1.5)	3.80E-04	7.64E-03
**PTRF**	1.5 (1.4-1.7)	1.10E-14	1.69E-02	1.3 (1.1-1.5)	6.50E-04	3.48E-02
**RAB27A**	1.6 (1.4-1.8)	6.90E-14	2.32E-04	1.3 (1.1-1.5)	5.20E-03	1.71E-02
**SCO1**	1.5 (1.2-1.8)	9.50E-06	7.16E-04	1.4 (1.1-1.7)	1.40E-03	1.04E-02
**SERPINE1**	1.3 (1.2-1.5)	6.90E-08	1.76E-03	1.2 (1-1.4)	9.60E-03	4.26E-02
**SH3D21**	1.2 (1.1-1.4)	2.60E-03	2.81E-02	1.4 (1.2-1.7)	8.70E-04	7.87E-03
**SLC16A3**	1.4 (1.2-1.5)	2.70E-08	4.85E-02	1.4 (1.1-1.7)	8.80E-04	9.17E-04
**SLC20A1**	1.3 (1.2-1.4)	1.20E-09	1.65E-02	1.3 (1.1-1.6)	1.10E-03	8.83E-03
**SLC43A3**	1.3 (1.2-1.4)	6.30E-07	1.00E-06	1.3 (1.1-1.6)	2.00E-03	2.26E-02
**SLC5A3**	1.2 (1-1.3)	7.00E-03	7.84E-03	1.3 (1.1-1.5)	4.30E-03	4.59E-02
**SMAGP**	1.8 (1.5-2)	0.00E+00	1.25E-03	1.3 (1.1-1.6)	2.40E-03	1.05E-02
**SOAT2**	1.4 (1.2-1.7)	3.10E-05	7.92E-03	1.3 (1.2-1.6)	1.40E-04	3.62E-03
**SOCS3**	1.4 (1.2-1.5)	3.60E-09	1.04E-02	1.4 (1.1-1.6)	4.90E-04	1.56E-02
**TBC1D10C**	1.5 (1.3-1.8)	2.00E-09	1.30E-05	1.3 (1.1-1.5)	3.90E-03	4.20E-03

**TIMP1**	1.5 (1.4-1.6)	0.00E+00	7.87E-04	1.4 (1.2-1.7)	4.70E-05	1.06E-02
**TMEM2**	1.5 (1.3-1.7)	2.40E-13	6.64E-03	1.3 (1.1-1.5)	9.00E-03	7.73E-03
**TMPRSS3**	1.2 (1.1-1.4)	2.20E-06	1.69E-03	1.2 (1.1-1.5)	6.30E-03	1.55E-02
**TNFSF14**	1.2 (1.1-1.3)	1.90E-08	1.53E-03	1.3 (1.1-1.5)	3.70E-03	3.32E-02
**TRAM2**	1.1 (1.1-1.2)	1.10E-04	6.56E-03	1.3 (1.1-1.5)	8.10E-03	2.10E-02
**TREML2**	1.2 (1.1-1.4)	9.00E-04	6.61E-04	1.3 (1.1-1.5)	6.80E-03	4.38E-02
**TSHZ2**	1.4 (1.2-1.7)	1.80E-06	4.40E-03	1.4 (1.1-1.8)	2.70E-03	8.83E-04
**TUBA1C**	1.7 (1.5-1.9)	0.00E+00	0.00E+00	1.4 (1.1-1.7)	2.80E-03	4.46E-02
**VDR**	1.5 (1.4-1.7)	4.40E-16	8.64E-04	1.2 (1.1-1.4)	3.80E-03	3.18E-02
**VNN3**	1.2 (1.1-1.4)	2.80E-04	9.13E-03	1.2 (1.1-1.4)	7.20E-03	5.91E-03

CI: confidence interval.

Before and after median OS: comparing gene expression level of patients who were dead before the median OS to ones who were dead after the median OS.

Median OS: GBM is approximately at 450 days (~15 months); LGG is approximately at 2700 days (~90 months).
